# Adaptive Edge-Response-Based Subpixel Localization Method for Microscopic Vision-Based Alignment Measurement

**DOI:** 10.3390/s26134040

**Published:** 2026-06-25

**Authors:** Xuefeng Sun, Weibo Wang

**Affiliations:** 1 Ultra-Precision Optoelectronic Instrument Engineering, Harbin Institute of Technology, Harbin 150080, China; 21b301009@stu.hit.edu.cn; 2 The Key Lab of Ultra-Precision Intelligent Instrumentation, Ministry of Industry and Information Technology, Harbin Institute of Technology, Harbin 150080, China; 3 Zhengzhou Advanced Research Institute of Harbin Institute of Technology, Zhengzhou 450001, China

**Keywords:** microscopic vision-based alignment, subpixel localization, optical measurement, edge-response modeling

## Abstract

Microscopic vision-based alignment measurement is a key procedure in micro-/nanoscale positioning, and its measurement repeatability mainly depends on the stability of subpixel edge–center estimation. However, in practical microscopic imaging, defocus and contamination can cause edge broadening and pseudo-gradient peaks, making it difficult for conventional methods to accurately estimate the edge center of alignment marks. To address this problem, this paper proposes an adaptive edge-response modeling method. First, an amplitude function is constructed by combining the gradient peak and the slope of the edge-transition region, enabling adaptive adjustment of the response amplitude and suppressing its coupling with other parameters. On this basis, the proposed model overcomes the limitation that the Sigmoid model is only suitable for single-edge fitting and enables unified modeling of practical multi-edge hybrid bonding marks. It also suppresses the interference caused by edge pseudo-peaks and abrupt gradient variations, thereby improving the accuracy of subpixel fitting and localization. Experimental results show that, compared with conventional methods, the proposed method improves the repeatability of subpixel edge localization under degraded microscopic imaging conditions by approximately 52%, meeting the requirements of high-precision microscopic vision-based alignment.

## 1. Introduction

With the continuous development of advanced semiconductor packaging toward high integration density and micro-/nanoscale manufacturing, high-precision microscopic vision-based alignment systems have become increasingly important in wafer bonding, chip interconnection, and micro-/nanostructure fabrication. In high-density advanced packaging, the alignment accuracy at the bonding interface directly affects the yield of multilayer integrated chips [1,2,3]. Therefore, higher requirements have been placed on the edge-resolution capability and measurement repeatability of microscopic vision-based alignment systems. Compared with conventional contact measurement methods, microscopic optical measurement offers the advantages of non-contact operation, high throughput, and suitability for complex micro-/nanostructure inspection. It has therefore become one of the key measurement techniques in advanced packaging and precision manufacturing [4,5].

As microscopic vision-based alignment measurement advances from micrometer-level to nanometer-level precision, current nanoscale alignment requirements place higher demands on the edge-resolution capability of microscopic imaging systems and the stability of subpixel edge-response models [6,7,8]. Conventional measurement models based on ideal edge assumptions can no longer meet the requirements of complex microscopic imaging conditions. In recent years, imaging degradation compensation, degradation-model constraints, and computational imaging methods based on microscopic images have been widely used to improve the spatial resolution and edge sharpness of microscopic imaging systems [9,10,11]. These methods can improve the edge distinguishability of alignment marks to some extent. However, for high-precision optical microscopic alignment measurement, improving spatial resolution alone cannot fundamentally recover the true optical response of degraded edges. It may also lead to inconsistencies between the reconstructed results and the real edge structures of the sample, thereby reducing the accuracy of optical microscopic alignment measurement [12,13,14].

High-precision edge measurement methods in current microscopic vision-based alignment systems mainly include template matching, feature matching, and subpixel measurement methods based on edge-response modeling [15,16]. Template matching methods usually estimate the position of alignment marks using normalized cross-correlation (NCC), sum of squared differences (SSD), and subpixel interpolation [17,18]. These methods are simple to implement and have relatively high computational efficiency. Feature matching methods usually perform local position estimation based on edges, corners, and regional structural features, and can adapt to certain rotation and scale variations. In contrast, subpixel edge measurement methods based on parameterized edge-transition models focus more on the optical transition characteristics of continuous edge responses [19]. By establishing a parameterized relationship between edge intensity response and spatial position, these methods enable subpixel edge measurement in microscopic alignment systems.

To achieve high-precision edge measurement in microscopic vision-based alignment systems, early studies commonly used continuous edge models, such as hyperbolic tangent functions, error functions (Erf), and arctangent functions, to parameterize the gray-level transition process of ideal edges. The subpixel edge position was then solved using least-squares optimization. Subsequently, Gaussian-blurred edge models were combined with edge-gradient analysis to describe the edge diffusion process under optical blur, enabling subpixel estimation of degraded edge responses [20,21,22,23]. In addition, some studies used two-dimensional edge discretization, normal-direction gradient modeling, and higher-order function approximation to locally reconstruct complex edge responses, thereby improving the stability of microscopic vision-based alignment measurement under non-ideal edge conditions [24]. However, when local abnormal responses or noise-induced pseudo-peaks occur at the edge, conventional models are prone to edge–center shifts, which reduces the repeatability and localization stability of microscopic optical measurement.

As discussed above, imaging degradation in microscopic vision alignment systems causes the edge response to deviate from the ideal step model. However, most existing subpixel edge measurement methods assume a continuous and monotonic ideal edge response, making it difficult to accurately characterize the optical properties of real degraded edges. Local abnormal responses or noise-induced spurious peaks can shift the estimated edge center, thereby reducing the repeatability and localization stability of microscopic optical measurements.

To address the above challenges, this paper proposes an adaptive edge-response modeling method for microscopic vision-based alignment measurement. Starting from the imaging degradation characteristics of degraded microscopic edges, the proposed method constructs an adaptive response amplitude function by combining the local gradient peak and the slope of the edge-transition region, thereby reducing the parameter coupling among amplitude, slope, and edge center. In addition, the single-edge Sigmoid transition model is extended to a multi-edge optical response model. Experimental results show that the proposed method achieves more stable subpixel edge–center estimation under degraded microscopic imaging conditions and improves the repeatability of microscopic vision-based alignment measurement.

The main contributions of this paper are summarized as follows:

- The subpixel edge measurement problem in microscopic alignment is reformulated from the perspective of imaging degradation and described as an edge-response characterization problem for degraded microscopic edges.
- An adaptive edge-response model is designed. By introducing a response amplitude model, the parameter coupling in conventional parameterized edge models is reduced, thereby improving the modeling stability under degraded edge conditions.
- The Sigmoid-based edge-transition model is extended from single-edge fitting to unified multi-edge modeling for practical bonding alignment marks.

The remainder of this paper is organized as follows. Section 2 describes the main concepts and theoretical foundations of the proposed method in detail. Section 3 presents and discusses the experimental results through qualitative and quantitative analyses and further validates the effectiveness of the proposed method through practical application tests. Finally, Section 4 summarizes the main findings of this work.

## 2. Materials and Methods

### 2.1. Imaging Degradation in Microscopic Vision-Based Alignment Systems

**Figure 1:**
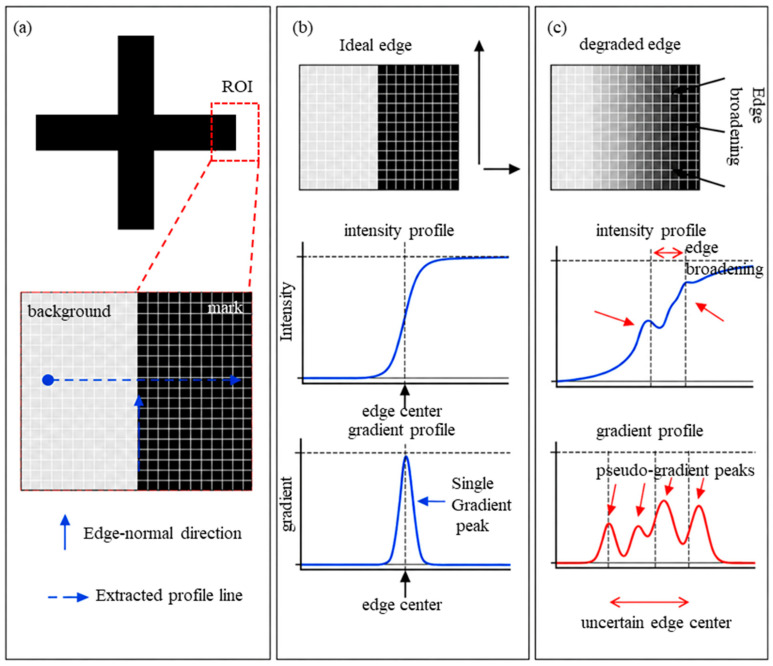
Schematic illustration of alignment mark edge responses in microscopic vision-based alignment measurement. (**a**) Schematic diagram of the alignment mark edge. (**b**) Ideal edge response of a microscopic vision-based alignment mark. (**c**) Practical edge response of a microscopic vision-based alignment mark.

In microscopic vision-based alignment measurement, the edges of alignment marks are the main information source for subpixel center estimation. As shown in Figure 1a, a one-dimensional intensity profile is usually extracted along the normal direction of the mark edge, and the subpixel edge position is determined from the edge response. Under ideal conditions, the boundary between the mark and the background can be approximated as a step-like intensity transition, and its gradient response exhibits a single dominant peak near the true edge center, as shown in Figure 1b. Therefore, conventional methods usually estimate the edge center based on the maximum gradient response or a predefined edge-transition model.

However, in practical microscopic vision-based alignment systems, the observed edge response is not an ideal step transition. Due to factors such as the limited numerical aperture of the objective lens, defocus, non-uniform illumination, and local reflection variations on the mark surface, an ideal edge is degraded into a microscopic edge response with a finite transition width. This process can be approximately expressed as:

(1) $$\hat{I}(x,y)=I(x,y)⊗PSF(x,y)+\varepsilon (x,y)$$

where x and y denote the pixel coordinates in the mark ROI image with a size of M × N. $\hat{I}(x)$ represents the degraded microscopic edge intensity response, I(x) represents the ideal edge intensity response, PSF denotes the point spread function of the microscopic imaging system, and ε(x) represents noise and local abnormal responses. The symbol ⊗ denotes the convolution operation. The convolution with the point spread function smooths the intensity variation and leads to broadening of the edge-transition region.

Under degraded microscopic vision imaging conditions, the edge response is also affected by local contamination, material-reflection variations, surface defects, and background interference, resulting in abnormal intensity fluctuations near the edge-transition region. These abnormal responses may form pseudo-gradient peaks in the gradient distribution, causing the maximum gradient position to deviate from the true edge center, as shown in Figure 1c. In addition, defocus and local reflection non-uniformity can cause asymmetric distortion of the edge-transition curve, making the edge center difficult to determine stably from a single gradient peak.

For conventional parameterized edge models, the edge center is usually estimated together with the response amplitude and edge slope. When the edge response is distorted, parameter coupling can easily occur among the amplitude, slope, and edge center. Local amplitude variations or edge-slope disturbances may cause shifts in the estimated edge center, thereby reducing the repeatability and stability of subpixel alignment measurement. Therefore, a subpixel measurement method for degraded microscopic edges should start from the response characteristics of microscopic edges, adaptively constrain the response amplitude, and establish a more stable edge–center estimation model.

### 2.2. Adaptive Edge-Response Modeling Method

**Figure 2:**
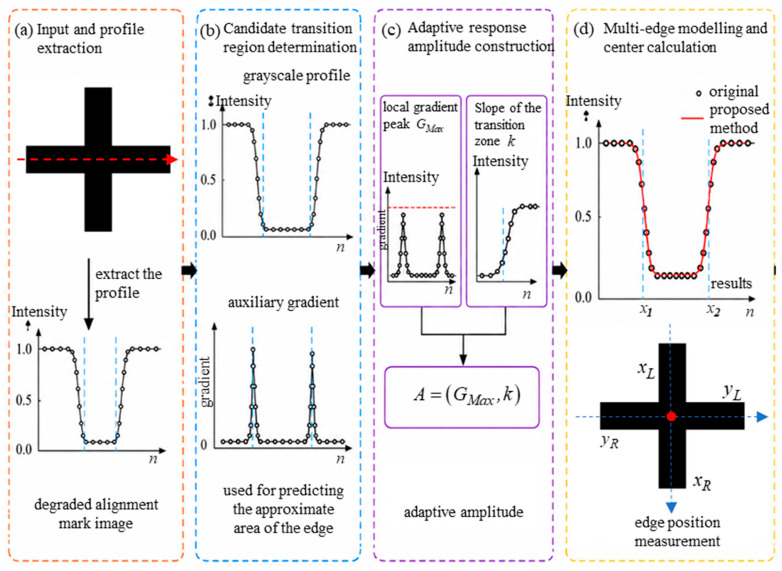
Schematic of the proposed adaptive edge-response modeling method. (**a**) Input image and one-dimensional profile extraction. (**b**) Determination of candidate transition regions. (**c**) Construction of adaptive response amplitudes. (**d**) Multi-edge modeling and center calculation.

Based on the analysis of the imaging degradation characteristics of degraded microscopic edges in Section 2.1, this paper further designs an adaptive edge-response modeling method to achieve stable estimation of the edge centers of microscopic alignment marks. As shown in Figure 2, the proposed method constructs an adaptive amplitude function by combining the edge-gradient peak and the slope of the edge-transition region. In this way, the response amplitude is transformed from a fully free fitting parameter into an adaptive term constrained by the local edge response, thereby reducing the parameter coupling among amplitude, slope, and edge center. Considering the high-precision monotonic representation capability of the Sigmoid function, a multi-edge Sigmoid-type edge-response model is further designed to uniformly describe multiple degraded edge intensity responses in complex alignment marks. This enables stable subpixel edge measurement under degraded microscopic imaging conditions.

For microscopic mark images, edge positions are mainly located in regions with abrupt intensity changes. To describe the optical edge response, this paper extracts a one-dimensional intensity profile along the x-direction of the mark:

(2) $$p\left(x\right)=\frac{1}{N}\sum_{y=1}^{N}I(x,y)$$

where x=1,2,…,M. Under ideal conditions, the edge-transition regions in the intensity profile $p\left(x\right)$ can be approximated as monotonic transitions, and their gradient responses should exhibit a single peak. However, under degraded microscopic imaging conditions, local reflection non-uniformity, defocus, defects, and contamination can cause profile broadening or pseudo-gradient peaks. In this case, methods that rely only on the maximum gradient or a fixed-form edge detection model are easily affected by local abnormal responses, resulting in edge–center shifts.

### 2.3. Parameterized Edge-Transition Model and Its Limitations

For estimating the center position of edge-transition regions in microscopic alignment marks, conventional methods are easily affected by edge-intensity fluctuations and pseudo-peaks under local reflection non-uniformity, defocus, defects, and contamination, resulting in shifts in subpixel position estimation. In contrast, the Sigmoid edge-response model can parameterize the overall dark-to-bright transition process of the edge-transition region, thereby providing a stable representation of the edge transition and enabling more robust edge–center estimation. The Sigmoid edge-transition response model can be expressed as:

(3) $$p\left(x\right)=A\times \frac{1}{1+e^{\left(-k(x-x_{0})\right)}}+B(x)$$

where *A* denotes the edge-response amplitude, *B* denotes the background term, k represents the edge slope, and $x_{0}$ represents the edge–center position. According to the first-order derivative, $p\left(x\right)$ remains strictly monotonic over the whole interval, and its derivative has a unique extremum. Therefore, the Sigmoid model can reduce, to some extent, the influence of complex optical microscopic imaging conditions on edge–center estimation.

However, the Sigmoid model is mainly designed for single-edge modeling. In practical microscopic alignment marks, edges usually appear in pairs or multiple pairs, making this model difficult to directly apply to the current subpixel estimation of edge–center positions. In addition, under degraded microscopic edge conditions, the response amplitude A, transition slope k, and edge center $x_{0}$ in the Sigmoid model are prone to parameter coupling. When the edge contrast decreases, or the transition region broadens, the model may adjust *A* and k, which can further cause a shift in $x_{0}$. Therefore, although the Sigmoid model has good capability for describing edge transitions, its single-edge form and free parameters still limit its application.

### 2.4. Adaptive Response Amplitude

To reduce the coupling among amplitude, slope, and edge center in the parameterized edge-response model, this paper transforms the edge-response amplitude from a fully free fitting parameter into an adaptive term constrained by the local edge response. Specifically, to suppress noise-induced peaks, only candidate peaks satisfying $g[x_{i}]>T_{G}$ are retained, where $T_{G}$ denotes an adaptive threshold calculated from the grayscale signal of the alignment mark:

(4) $$T_{G}=\xi \cdot 1.4826\cdot median(|G[i]-median(G)]|)$$

The coefficient *ξ* is typically set within the range of 1–5. The factor 1.4826 converts the median absolute deviation into an equivalent standard deviation, assuming that the noise approximately follows a Gaussian distribution. The discrete gradient is subsequently calculated using the central-difference scheme:

(5) $$G[x]=\frac{p[x+1]-p[x-1]}{2\Delta x}$$

where G[x] denotes the discrete gradient. Here, x represents the discrete position in the intensity profile, and G[x] describes the intensity variation rate at position x. Its magnitude is denoted as g[x]=|G[x]|.

Next, the true mark edges need to be determined. Since pseudo-peaks in the discrete signal can directly affect the estimation of $G^{peak}$, the true edge set of the mark should be identified from g[x] for subsequent adaptive parameter estimation.

(6) $$P=\left\{x_{i}|g[x_{i}]>g[x_{i}-1]\&g[x_{i}]>g[x_{i}+1]\right\}$$

Since the structure of the hybrid bonding alignment mark is predefined, the true edge peaks appear as a sparse and ordered distribution in the gradient response. To suppress the interference of pseudo-peaks, any two detected peaks are also required to satisfy a minimum spacing constraint. Finally, the peak indices are sorted according to the known structure of the hybrid bonding alignment mark to obtain the final edge-peak indices.

The gradient peak at the true edge index $x_{i}$ is denoted as $G_{i}^{peak}$. To reduce the influence of discrete sampling on peak estimation, three-point quadratic interpolation is used to estimate the subpixel peak height:

(7) $$G_{i}^{peak}=g[x_{i}]-\frac{{\left(g[x_{i}-1]-g[x_{i}+1]\right)}^{2}}{8(g[x_{i}-1]-2g[x_{i}]+g[x_{i}+1])}$$

$G_{i}^{peak}$ denotes the local gradient peak amplitude of the *i*-th edge, which is used to characterize the actual optical response strength of this edge. In the adaptive Sigmoid-based multi-edge model, the transition response of the *i*-th edge can be expressed as:

(8) $$p_{i}(x)=\frac{A_{i}}{1+e^{\left(-k_{i}(x-x_{i})\right)}}$$

(9) $$p_{i}{\left(x\right)}^{’}=A_{i}\cdot \frac{k_{i}e^{\left(-k_{i}(x-x_{i})\right)}}{{\left(1+e^{\left(-k_{i}(x-x_{i})\right)}\right)}^{2}}$$

When $x=x_{i}$, the slope of the Sigmoid transition response reaches its maximum, and the corresponding maximum gradient response is given by:

(10) $$\max(p_{i}{\left(x\right)}^{’})=\frac{\left|A_{i}\right|k_{i}}{4}$$

To make the maximum edge response of the model consistent with the local gradient peak of the real microscopic edge, this paper defines:

(11) $$\max(p_{i}{\left(x\right)}^{’})=G_{i}^{peak}$$

Thus, the adaptive response amplitude of the i-th edge can be obtained as:

(12) $$A_{i}=\frac{4G_{i}^{peak}}{k_{i}}$$

With the above constraint, the response amplitude $A_{i}$ is jointly determined by the local gradient peak of the real edge and the transition slope of the model, rather than being estimated as a fully free parameter. This effectively reduces parameter coupling. It should be noted that the gradient peak $G_{i}^{peak}$ is used only as an adaptive amplitude parameter in this paper, rather than as an exact physical quantity.

### 2.5. Sigmoid-Based Multi-Edge Transition Response Modeling

Practical optical microscopic alignment marks usually contain multiple symmetric edges, such as cross-shaped and rectangular structures, where the mark center is jointly constrained by multiple edges. To improve the robustness of subpixel position estimation for complex alignment marks, this paper extends the single-edge Sigmoid model to a multi-edge transition response model. The multi-edge transition response can be expressed as:

(13) $$p(x)=\sum_{i=1}^{N}\left[\frac{A_{2i-1}}{1+e^{-k_{2i-1}(x-x_{2i-1})}}-\frac{A_{2i}}{1+e^{-k_{2i}(x-x_{2i})}}\right]+B(x_{i})$$

where $℧=\overset{N}{\underset{i}{\cup }}\left[x_{i}-W_{i},x_{i}+W_{i}\right]$ denotes the effective fitting region, and $W_{i}$ represents the width of the effective edge-transition region of the *i*-th edge, *N* is a known constant, and both the edge spacing and the minimum peak height are predetermined. The fitting region $W_{i}$ is determined by searching outward from the gradient peak toward both sides, with the positions at which the intensity variation becomes stable defined as its boundaries. This ensures that $W_{i}$ contains the complete edge-transition region and the stable intensity plateaus on both sides, thereby satisfying the step-like response required for Sigmoid modeling. In this study, the boundary range of $W_{i}$ was set to 10–20 pixels.

Through the above multi-edge transition response modeling, the proposed method can simultaneously use multiple edge-structure constraints in the alignment mark, thereby reducing the position estimation shift caused by local abnormal edge responses. The pseudocode of the complete algorithm is presented in Algorithm 1.
**Algorithm 1. Adaptive Edge-Response Modeling Method**InputOne-dimensional intensity profile (p(x))1:Extract the one-dimensional intensity profile (p(x)) along the edge-normal direction.2:Smooth (p(x)) and calculate its gradient response (g(x)).3:Detect candidate edge locations ($x_{i}$) from the local extrema of (g(x)).4:Calculate the local gradient peak ($G_{i}^{peak}$).5:Estimate the adaptive amplitude ($A_{i}=\left\{G_{i}^{peak},k_{i}\right\}$) and construct the multi-edge response model.6:Optimize the model parameters and obtain the subpixel edge positions ($x_{i}$).OutputSubpixel edge positions ($x_{i}$)

## 3. Results

### 3.1. Experimental System

**Figure 3:**
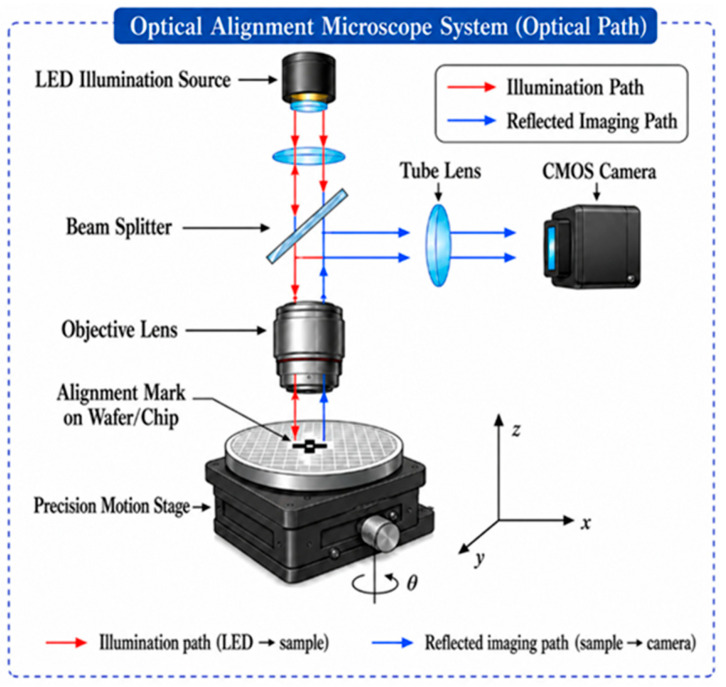
Optical path diagram of the microscopic vision-based alignment system.

**Figure 4:**
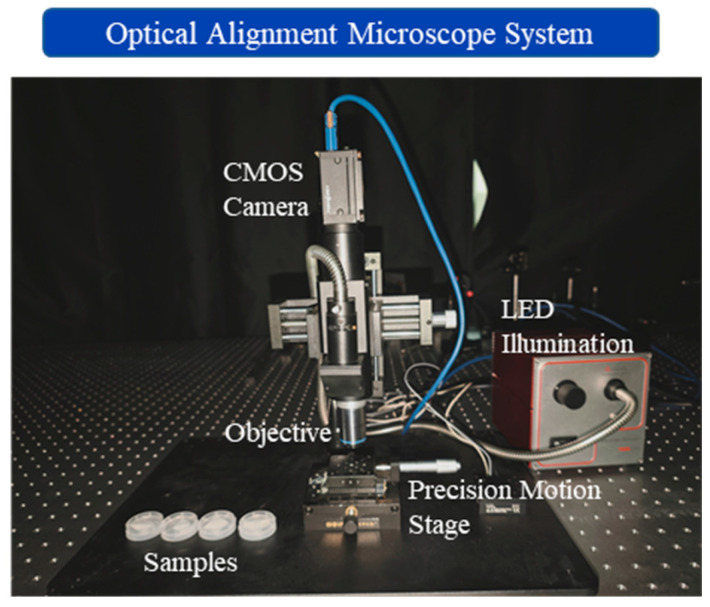
Experimental microscopic vision-based alignment system.

To verify the effectiveness of the proposed adaptive edge-response modeling method in microscopic vision-based alignment measurement, an optical imaging experimental system for alignment marks was established, and experiments were conducted on various wafer-level hybrid bonding alignment marks. The system mainly consists of a microscope objective, a reflected narrow-band illumination source, optical filters, a CMOS industrial camera, and a nanometer-scale displacement stage, as shown in Figure 3 and Figure 4.

In the experiment, a CMOS industrial camera was used to acquire alignment mark images, with an image resolution of 2448 × 2048, a pixel size of 3.45 μm, and a frame rate of 75 fps. The imaging system employs a 630 nm illumination source and 20× and 40× objective lenses. To obtain a controllable displacement reference, a nanometer-scale displacement stage with a full-stroke repeatability of ±3 nm was introduced into the system. It was used to conduct small step–displacement and reciprocating–displacement experiments, and to evaluate the localization accuracy and alignment measurement repeatability of different methods under practical displacement conditions.

In addition, an Olympus OLS5000 microscope (Olympus Corporation, Tokyo, Japan) was used to construct mark imaging experiments under different defocus conditions, aiming to verify the robustness of the proposed method against edge broadening and optical response degradation caused by defocus. The experimental evaluation mainly included edge–center estimation stability, controllable displacement measurement error, alignment repeatability expressed by 3σ, and computational efficiency.

### 3.2. Evaluation Metrics

In this paper, 3σ repeatability is used as the main evaluation metric for the stability of microscopic vision-based alignment measurement. For the same alignment mark, images are repeatedly acquired under the same imaging conditions, and the corresponding alignment positions are calculated, yielding *S* measurement results $x_{i}$. The standard deviation is expressed as:

(14) $$\sigma =\sqrt{\frac{1}{S-1}\sum_{i=1}^{S}{\left(x_{i}-\bar{x}\right)}^{2}}$$

Here, *x* denotes the mean position obtained from 50 repeated measurements. Each simulated hybrid bonding alignment-mark group contained 30 samples, while the real hybrid bonding alignment-mark experiment included 10 samples. The corresponding alignment measurement repeatability is defined as 3σ [25]. A smaller 3σ value indicates lower position fluctuation in repeated measurements and more stable subpixel edge estimation.

In this study, blurred, contaminated, and defective edges were generated from an ideal step edge. The blurred edges were produced using Gaussian filtering with blur levels ranging from 0 to 10. The contaminated edges were simulated by superimposing local intensity disturbances near the edge, whereas the defective edges were generated by introducing local notches or structural loss. These degradation models were used to approximate defocus, contamination, and edge damage in practical microscopic imaging.

### 3.3. Validation Using Simulated Microscopic Degraded Edge Responses

**Figure 5:**
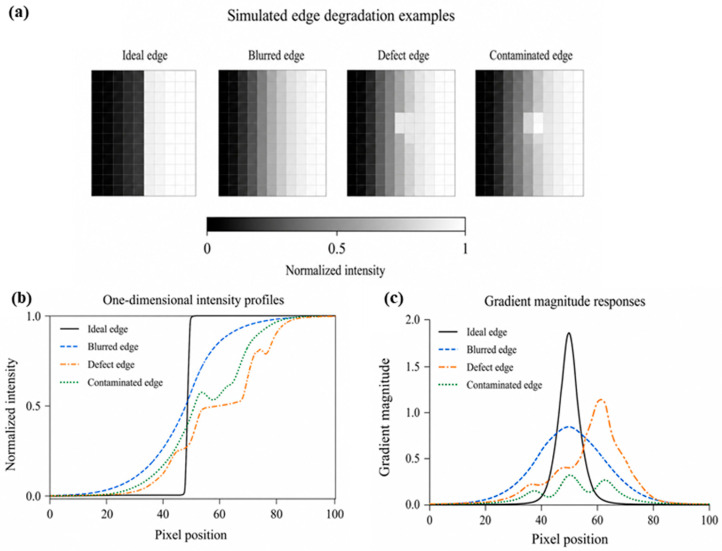
Simulated degraded edge responses in optical microscopic imaging. (**a**) Simulated optical microscopic degraded edge images, including ideal, blurred, defective, and contaminated edges. (**b**) One-dimensional intensity profiles corresponding to different degraded edges. (**c**) Gradient response curves corresponding to different degraded edges.

To verify the modeling capability of the proposed method for degraded edges in optical microscopic alignment mark images, typical degraded edge responses, including ideal edges, blurred edges, defective edges, and contaminated edges, were constructed. As shown in Figure 5a, the ideal edge exhibits a clear intensity step, whereas the blurred edge shows obvious transition broadening due to equivalent optical diffusion. The defective and contaminated edges introduce local structural disturbances and abnormal intensity responses, respectively, resulting in asymmetric variations and local pseudo-responses in the edge-transition region.

The corresponding one-dimensional intensity profiles and gradient responses are shown in Figure 5b,c. The ideal edge has a clear intensity step and a single gradient peak, whereas the degraded edges exhibit edge-transition broadening, local abnormal responses, and pseudo-gradient peaks, making it difficult to stably estimate the true edge center.

**Figure 6:**
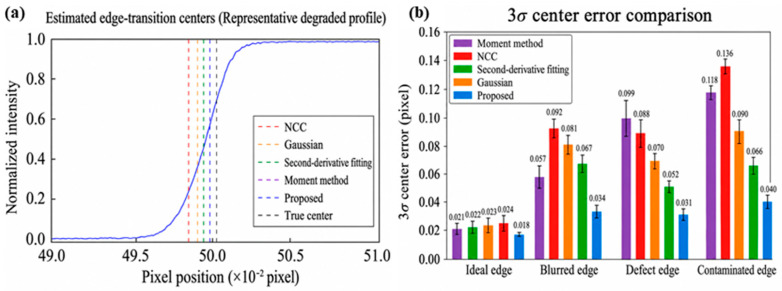
Comparison of edge−center estimation results under degraded edge conditions. (**a**) Edge–center estimation results of different methods for representative degraded edges. (**b**) Comparison of 3σ center errors of different methods for standard, defocused, contaminated, and defective images.

Figure 6a further compares the edge–center estimation results of different methods for the simulated degraded optical microscopic alignment mark edges. It can be observed that the edge centers estimated by all methods are generally concentrated near the true edge center, but local deviations of approximately 0.1–0.2 pixels still exist. Among them, NCC [26], the Gaussian method [25], second-derivative fitting [27], and the moment method [15,24] are more easily affected by local intensity fluctuations and gradient peak variations, resulting in different degrees of estimation shifts. In contrast, the proposed method shows higher accuracy and better anti-interference stability.

Figure 6b presents the repeatability comparison results for standard, defocused, contaminated, and defective edges. For the standard edge, all methods achieve relatively small errors. Except for the proposed method, the 3σ repeatability errors of the other methods are mainly in the range of 0.021–0.024 pixels, whereas that of the proposed method is approximately 0.018 pixels, corresponding to an improvement of approximately 16.67–33.27%. This improvement becomes more significant for defocused, contaminated, and defective edges. These results indicate that the proposed method provides better center-estimation stability under simulated degraded optical microscopic alignment mark edge conditions.

**Figure 7:**
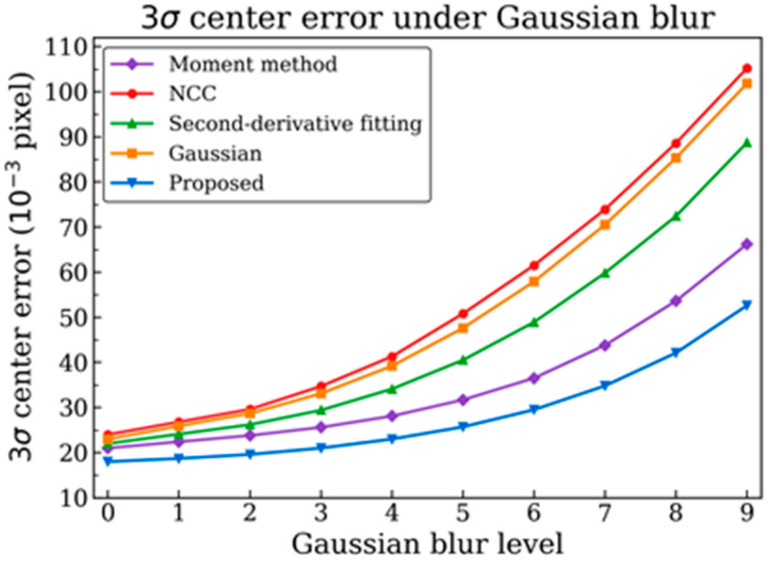
Variation in edge–center estimation error of different methods under different Gaussian degradation intensities.

Figure 7 shows the variation in edge−center estimation error of different methods under different Gaussian blur degradation intensities. As the blur intensity increases, the 3σ center errors of all methods gradually increase, but their growth rates are clearly different. Under low-blur conditions, the errors of different methods are relatively close. However, when the blur intensity becomes stronger, the errors of NCC and the Gaussian method increase more significantly, indicating that these methods are more sensitive to edge-intensity broadening. At the maximum blur intensity, the errors of conventional methods increase markedly, whereas the proposed method still maintains the lowest error, reducing the error by approximately half compared with NCC and the Gaussian method. These results indicate that the proposed method can maintain stable center-estimation capability when the edge-transition region is significantly broadened, verifying the effectiveness of adaptive edge-response modeling for blurred, degraded microscopic edges.

### 3.4. Real Microscopic Mark Measurement Experiment

**Figure 8:**
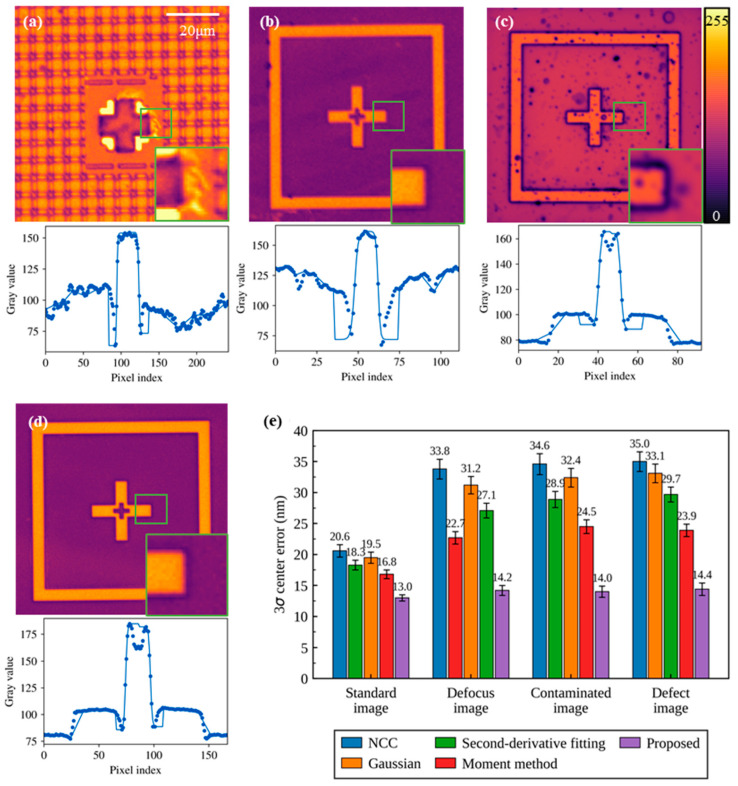
Measurement results for real degraded optical microscopic alignment mark images. (**a**–**d**) Real degraded optical microscopic mark images with ideal, blurred, defective, and contaminated edges, respectively. The edge-fitting curve obtained by the proposed method is shown below each image. (**e**) Comparison of 3σ center errors of different methods for standard, defocused, contaminated, and defective images.

**Figure 9:**
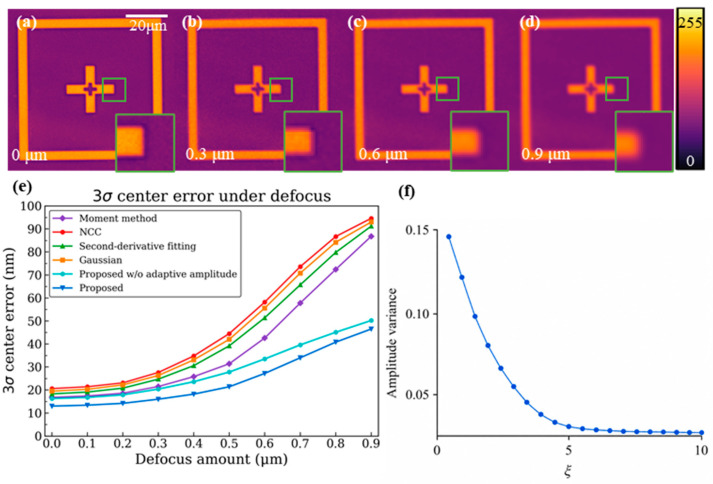
Edge–center estimation stability under defocused conditions. (**a**–**d**) Defocused optical microscopic alignment mark images, where the focal plane is set to 0 μm, the defocus step is 0.1 μm, and the maximum defocus distance is 0.9 μm. (**e**) Stability analysis of edge–center estimation using different methods under defocused conditions. (**f**) Effect of the noise suppression coefficient ξ on response amplitude prediction.

To verify the effectiveness of the proposed method in practical microscopic vision-based alignment measurement, standard, defocused, contaminated, and defective images were selected for comparative experiments. The real microscopic images shown in Figure 8a–d indicate that the standard image has a relatively clear edge transition, the defocused image exhibits obvious edge broadening, the contaminated image contains local abnormal intensity responses, and the defective image shows edge-structure damage and discontinuous responses. These degradation factors weaken the stability of edge-gradient peaks and increase the difficulty of edge–center estimation. In addition, the 3σ center error was used to evaluate measurement repeatability. As shown in Figure 9, under standard image conditions, the 3σ error of the proposed method is 13.0 nm, which is lower than the 16–21.0 nm obtained by other methods. The average error is reduced by approximately 30% compared with conventional methods, indicating that the proposed method still provides higher center-estimation stability under clear edge conditions.

For the defocused, contaminated, and defective images, the errors of conventional methods increase significantly and are mainly distributed in the range of 20–35.0 nm, whereas the proposed method remains at approximately 14 nm. Taking the contaminated and defective images as examples, the 3σ errors of the proposed method are 14.0 nm and 14.4 nm, respectively, which are approximately 55–60% lower than those of NCC and the Gaussian method. These results indicate that the proposed method can effectively suppress the influence of local abnormal responses, pseudo-gradient peaks, and edge-structure damage on center estimation, thereby improving the repeatability of alignment measurement in real degraded microscopic images.

As the defocus distance increases from 0 μm to 0.9 μm, the variations in 3σ center error are compared among NCC, the Gaussian method, second-derivative fitting, the moment method, the proposed method without adaptive amplitude, and the complete proposed method. The results show that when the defocus distance is small, the errors of all methods increase slowly. When the defocus distance exceeds 0.5 μm, the errors of conventional methods increase significantly, whereas the proposed method maintains a lower error and a smoother increasing trend. This indicates that the proposed adaptive edge-response modeling method has better defocus robustness and higher edge–center estimation stability. In addition, the effect of the noise suppression coefficient ξ on the stability of response amplitude prediction was investigated. As shown in Figure 9f, the normalized variance of the predicted amplitude decreases rapidly as ξ increases and gradually converges to approximately 0.026. When ξ is in the range of 3.5–5, noise interference is effectively suppressed, and the amplitude prediction becomes stable, thereby improving the repeatability and measurement accuracy of subpixel localization.

In addition to alignment accuracy, subpixel localization time is also an important indicator of the engineering applicability of hybrid bonding alignment systems. Experimental results show that the processing time for a single subpixel alignment measurement is less than 90 ms, which is significantly shorter than the second-level alignment time typically available in practical hybrid bonding equipment and therefore satisfies the real-time requirements of high-precision bonding alignment systems.

## 4. Conclusions

This paper proposes an adaptive edge-response modeling method to address the instability of subpixel edge–center estimation caused by degraded edges in microscopic vision-based alignment measurement. The proposed method constructs an adaptive response amplitude by combining the local gradient peak and the slope of the edge-transition region. In this way, the edge-response amplitude can be adjusted according to the local degree of degradation, thereby reducing the parameter coupling among amplitude, slope, and edge center in conventional parameterized edge models. This improves the stability of edge–center estimation under complex imaging conditions.

Experimental results show that the proposed method can effectively suppress the influence of edge broadening, pseudo-gradient peaks, and local abnormal responses caused by defocus, contamination, and defects. In simulated degraded edges, real microscopic images, and defocus experiments, the proposed method achieves lower 3σ center errors. In particular, the error reaches approximately 13.0 nm for standard images and remains at approximately 14.2 nm for defocused, contaminated, and defective images. The ablation experiment further shows that the model error increases after removing the adaptive amplitude constraint, verifying the effectiveness of this constraint in improving the stability of edge–center estimation.

Overall, the proposed method improves the repeatability and stability of microscopic vision-based alignment measurement under degraded edge conditions. It is suitable for high-precision alignment, precision inspection, and micro-/nanoscale vision measurement applications. However, the experiments in this paper are mainly conducted under typical degradation conditions, including defocus, contamination, and defects, and the sample types and practical operating conditions are still limited. In addition, the edge localization accuracy may decrease when the edge-gradient response is excessively weak or when the actual edge response deviates substantially from the step-like response assumed by the proposed method. Future work will extend the validation to different materials, various alignment-mark structures, and more complex degradation scenarios. The computational efficiency of the algorithm will also be further improved to facilitate the industrial integration of the complete system and meet the real-time requirements of online measurement and industrial inspection applications.

## Data Availability

Data is provided within the manuscript.
